# Highly Water-Soluble Microneedle Patch for Short Wear Time and Rapid Drug Delivery

**DOI:** 10.1021/acs.molpharmaceut.4c01207

**Published:** 2024-12-03

**Authors:** Amy J. Wood-Yang, Abishek Sankaranarayanan, Max J. Freidlin, Mark R. Prausnitz

**Affiliations:** School of Chemical and Biomolecular Engineering, Georgia Institute of Technology, Atlanta, Georgia 30332, United States

**Keywords:** microneedle patch, rapid drug delivery, fast-dissolving formulation, lidocaine, effervescence, transdermal delivery

## Abstract

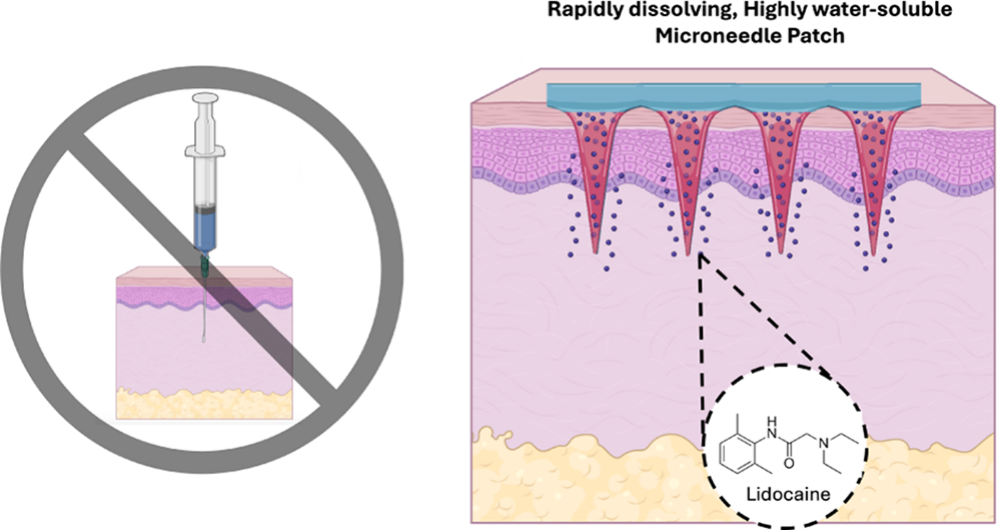

Treatment of acute medical conditions such as pain would benefit from rapid drug delivery and improved ease of administration of local anesthetics that currently have a slow onset of action by topical or oral administration and require expert administration by injection. To address this need, microneedle (MN) patches containing needlelike projections made from a polymer/drug matrix can be painlessly pressed into the skin for local or systemic drug delivery. To improve the speed and ease of drug delivery, we present a rapidly dissolving, highly water-soluble MN patch, which minimizes the wear time to 10 s to improve drug delivery in situations where rapid delivery with simplified administration is needed. MNs were made of polyvinylpyrrolidone (PVP), which is soluble in both water (enabling dissolution in the skin) and polar organic solvents (facilitating coformulation with lidocaine (L)). The addition of a highly water-soluble salt, sodium bicarbonate (NaB), to PVP/L MNs allowed for 60% faster MN dissolution in porcine skin ex vivo. Further addition of citric acid to generate effervescence upon reaction with NaB did not further decrease the MN dissolution time in the pig skin and led to poor shelf-life stability due to premature effervescence during storage. The PVP/L/NaB MNs delivered 23.8 ± 3.5 μg lidocaine to the skin ex vivo, well above the expected dose for local analgesic effect. Our highly water-soluble PVP/L/NaB MN design enables shorter wear time for faster delivery compared to the oral or topical route and easier administration compared to injection currently used for the delivery of drugs needing a rapid onset of action.

## 1 Introduction

There are many acute medical conditions where rapid drug delivery is needed, such as pain management.^1,2^ The oral and topical routes have slower drug absorption and action than injection routes,^3−5^ making injection attractive for drugs like local anesthetics.^6^ However, injection has several inherent disadvantages, such as the requirement for expertise in administration and generation of biohazardous sharps waste, pain, and needle phobia.^7,8^

Microneedle (MN) patches offer a solution to address the disadvantages of injecting local anesthetics. MN patches contain an array of needlelike projections less than 1 mm in height that are inserted into the skin as a method of drug delivery without the need for hypodermic needles. MNs do not generate biohazardous sharps waste, require minimal expertise to administer, and are associated with less pain and needle phobia compared to hypodermic injections.^9^ Dissolvable MNs detach from the patch backing upon insertion and dissolve in the viable epidermis and dermis while releasing the drug.^9−11^ However, faster MN dissolution would be desirable for acute medical conditions such as pain management. Faster MN dissolution could allow for faster drug release from the MN and, thus, faster onset of drug action. Additionally, faster MN dissolution could allow for a shorter patch wear time, which would be useful during an acute medical condition, especially if the patient is in discomfort or pain.

Our solution to increasing the dissolution speed of dissolving MNs for rapid drug delivery is adding water-soluble salts to the MN formulation. These excipients could act like disintegrants when the MNs are exposed to aqueous interstitial fluid when inserted into the skin. We also hypothesized that making the formulation effervescent could further speed up MN dissolution through gas bubble generation, similar to effervescent tablets.^12^ In the presence of water, effervescent MNs can rapidly react to generate a CO_2_ gas, which could facilitate MN disintegration.

Polyvinylpyrrolidone (PVP) can be used as the MN polymer matrix, which may be useful because it is soluble in both water and some organic solvents.^13^ Since water cannot be used during manufacturing of effervescent MNs (i.e., to avoid triggering the effervescent reaction during fabrication), PVP can allow for effervescent formulations to be incorporated into dissolving MNs with an organic solvent casting solution.

Our approach of using PVP and water-soluble salts is applicable to both water-soluble and poorly water-soluble drugs. Many of the water-soluble polymers commonly used in dissolvable MNs,^11,14^ such as poly(vinyl alcohol), gelatin, and carboxymethylcellulose, can only be formulated with drugs that are also soluble in an aqueous MN casting solution. By contrast, PVP can be formulated with poorly water-soluble drugs in an organic solvent casting solution while still allowing for fast dissolution in water.

The objective of this study was to design a MN patch comprising PVP, a water-soluble salt, and a drug for rapid drug delivery to minimize the patch wear time to 10 s. Lidocaine was chosen as a model drug that would be of interest for rapid delivery by MN patch for local anesthesia. The hypothesis of this study was that adding water-soluble salts to the MN casting formulation would speed up MN dissolution in the skin, thereby facilitating rapid drug delivery. We also hypothesized that making the water-soluble salt formulation effervescent (by adding sodium bicarbonate (NaB) and citric acid (CA) to the MN formulation) will further speed up dissolution. We evaluated MN patches in the pig skin ex vivo to compare dissolution kinetics and found that patches could deliver a therapeutically relevant lidocaine dose to the skin.

## 2 Materials and Methods

### 2.1 Materials

Lidocaine, NaB, sulfohodamine B (SRB), CA monohydrate, sodium phosphate dibasic anhydrous (NaP), and PVP (MW 10 kDa, 55 kDa, 360 kDa) were obtained from Sigma-Aldrich (St. Louis, MO). A Sylgard 184 silicone elastomer kit was obtained from Dow Corning (Midland, MI).

### 2.2 Microneedle Fabrication

Polydimethylsiloxane (PDMS) molds were made by mixing the 10:1 base:curing agent from the Sylgard 184 silicone elastomer kit and pouring the mixture over master structures of 112 microneedles arranged in a circular geometry of diameter 1.1 cm, as previously described.^15^ Each MN was conical in shape, measuring 600 μm in height with a 250 μm cone base diameter and a tip diameter of ∼10 μm. Each MN was positioned on top of a funnel-shaped pedestal of 600 μm base diameter, 250 μm top diameter, and 300 μm height

CA was dried in a vacuum oven at 60 °C and −93 kPa for two nights and then stored in a desiccator at room temperature (20–25 °C) to evaporate off the molecular water to increase stability. NaB and NaP were each finely ground using a mortar and pestle immediately before use. PVP was dissolved in ethanol (100%, anhydrous) while stirring at 40 °C, and 0.05% w/w SRB dye was added. For formulations containing CA, CA was added to the solution and vortexed. For some formulations, NaB or NaP was then added to the solution as a suspension and vortexed.

To make MNs, 70 μL of the formulation was immediately cast into PDMS molds at room temperature on a vacuum chuck with −95 kPa suction from the bottom of the PDMS mold for 5 min. Formulations used in this study are listed in Table 1. The PDMS mold was covered with a box while on the vacuum chuck to minimize ethanol evaporation. The PDMS molds were centrifuged at 3234 rcf for 2 min at room temperature. Then, 100 μL ethanol was added on top of the PDMS mold and a razor blade was used to push the ethanol into the funnel region of the mold cavities and scrape off excess casting solution. This was repeated two more times. Finally, 100 μL ethanol was added on top of the PDMS molds again and the molds were centrifuged for 2 min at 3234 rcf at room temperature.

**Table 1:** MN Formulations Used in this Study^a^

Formulation name	PVP 1:1:1 10:55:360kDa	Lidocaine	Sodium bicarbonate	Citric acid	Sodium phosphate
PVP	10	-	-	-	-
PVP/L	10	7.11	-	-	-
PVP/L/CA	10	7.11	-	24	-
PVP/L/NaB	10	7.11	30	-	-
PVP/L/NaB/CA^b^	10	7.11	30	24	-
PVP/L/NaP/CA	10	7.11	-	24	30

a All excipients are listed as % w/w.

b Effervescent formulation.

The PDMS molds were dried overnight on a 40 °C hot plate. The next day, the PDMS molds were placed on the vacuum chuck at room temperature with −95 kPa suction, and 100 μL of patch backing solution (13.5% w/w PVP (55 kDa), 13.5% w/w PVP (360 kDa) in ethanol) was cast on top for 30 min. Another 100 μL of backing solution was added for another 30 min. The PDMS molds were dried overnight on a 40 °C hot plate. The next day, UV-curable adhesive (RapidFix, LHB Industries, St. Louis, MO) was added on top of the dried patch backing, and an acrylic sheet (McMaster-Carr, Douglasville, GA) laser-cut into a 2 cm diameter disc was pressed on top of the glue. UV light was applied through the disc for 20 s to cure the glue and attach the patch backing to the disc. The MN patch was demolded and dried under a −93 kPa vacuum at 55 °C for 2 h. MN patches were stored in a desiccator at room temperature until use.

### 2.3 Determining Extent of MN Dissolution in the Porcine Skin Ex Vivo

Scissor-clipped porcine abdominal skin (Pel-Freez, Rogers, AR) was trimmed of excess fat and then incubated on top of a shallow bath of deionized (DI) water at room temperature for 2 h. The skin was then dried and stretched. The MN height was measured by optical microscopy (model SZX12, Olympus, Tokyo, Japan). MN patches were applied to the skin with a force of 66.8 ± 1.2 N and a velocity of 4.42 ± 0.54 m/s using an in-house-designed patch applicator and then held pressed into the skin for 10 s. MN patches were removed from the skin, and the height of the MNs was measured by microscopy. In some cases, the patches were pressed into the skin for 10 s and then left on the skin for a longer time of up to 1 min. Fluorescence microscopy images of the skin were taken immediately after patch removal (model SZX12, Olympus). The insertion site was then covered in gentian violet solution for 15 min, which was then wiped away by 70% isopropyl alcohol swabs (Becton Dickinson and Company, Franklin Lakes, NJ) to leave the sites of MN penetration stained.

The percentage of MN height dissolved after insertion was determined by eq 1.
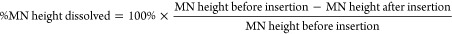
1

Three different MNs were measured on each patch, and the mean value was reported. Significance of changes in MN height was determined by one-way ANOVA with Tukey’s posthoc comparison.

### 2.4 MN Insertion into the Agarose Gel Skin Model

Agarose was dissolved in DI water at 0.0265 g/mL to mimic the viscoelastic properties of the skin.^16^ The agarose solution was fully dissolved by a hot water bath (90 °C), immediately poured into a container, left to cool. The gel was covered with a wet paper towel and stored at 4 °C until use. A single MN from a patch was taped to a holder, and a piece of agarose gel was inserted on top of the MN while being recorded by fluorescence or bright-field microscopy (model SZX12, Olympus).

### 2.5 Ambient Condition Stability Study

MN patches were left on the benchtop at room temperature and humidity (40–60% RH) for 24 h, and the same MN from each patch was imaged at time points by bright-field microscopy (model SZX12, Olympus). At least three different patches for each experimental group were imaged.

### 2.6 Fourier-Transform Infrared (FTIR) Spectroscopy

Powder or film samples were placed on a Shimadzu IRAffinity-1S FTIR spectrophotometer (Kyoto, Japan) at room temperature, and the light intensity was measured from 400 to 4000 cm^–1^ after background correction.

### 2.7 Thermogravimetric Analysis (TGA)

MN patches were broken into pieces (∼15 mg) and placed in sample pans with lids in a Mettler Toledo TGA 2 (Columbus, OH) to determine the moisture content of each patch. The patches were heated from 25 to 150 °C at 5 °C/min, and the percent original mass lost was measured. The percent water content by weight was taken as the difference in mass between 110 and 25 °C, since degradation of pure NaB appeared to occur above this temperature (Figure S1A).

### 2.8 High-Performance Liquid Chromatography (HPLC) Analysis of the Dose Delivered

After MN patch insertion into the skin and removal after 10 s, the used patches were dissolved in 1 mL of DI water for at least 15 min. The skin surface at the site of patch removal was wiped with an alcohol swab (Becton Dickinson and Company), and the swab was placed in a 1 mL solution of 1:1 acetonitrile:water to extract lidocaine collected from the skin surface. A 1 cm^2^ piece of skin encompassing the site of patch insertion was excised with a scalpel and cut into ∼15 smaller pieces before being placed in a 2 mL solution of 1:1 acetonitrile:water to extract lidocaine. The swab and skin in acetonitrile:water solutions were left to shake for 36 h.

Samples were spun down at 16 000 rcf for 10 min and then filtered with Acrodisc 13 mm Nylon 0.45 μm syringe filters (Cytiva, Marlborough, MA). Filtered samples were analyzed with a reversed-phase column (ZORBAX Eclipse XDB-C18, 5 μm, 4.6 mm × 150 mm, Agilent, Santa Clara, CA) in an Agilent 1260 Infinity II HPLC system. The mobile phase followed a linear gradient of 5:95 acetonitrile:water with 0.1% trifluoroacetic acid at 0 min and 60:40 acetonitrile:water with 0.1% trifluoroacetic acid at 5 min. The mobile phase flow rate was 1.25 mL/min, and lidocaine was detected at 254 nm with a constant column temperature of 25 °C. The lidocaine retention time was 4.27 min, as confirmed with commercial lidocaine (Sigma-Aldrich). Negative controls of PVP/NaB patches and skin not exposed to lidocaine were similarly prepared and analyzed. Samples exposed to skin had a slightly later lidocaine retention time of 4.3 min, so a secondary calibration curve was made using a diluent of acetonitrile:water that had been exposed to excised skin away from the site of patch insertion.

### 2.9 Film Casting of Formulations

To create films having the same composition as MNs, formulations from Table 1 were made, and 200 μL was cast onto a flat PDMS surface and dried on a 40 °C hot plate for ∼72 h. Images were taken with a Canon EOS 60D camera (Tokyo, Japan).

## 3 Results

### 3.1 MNs Made of PVP Enable Rapid MN Dissolution

**Figure 1:**
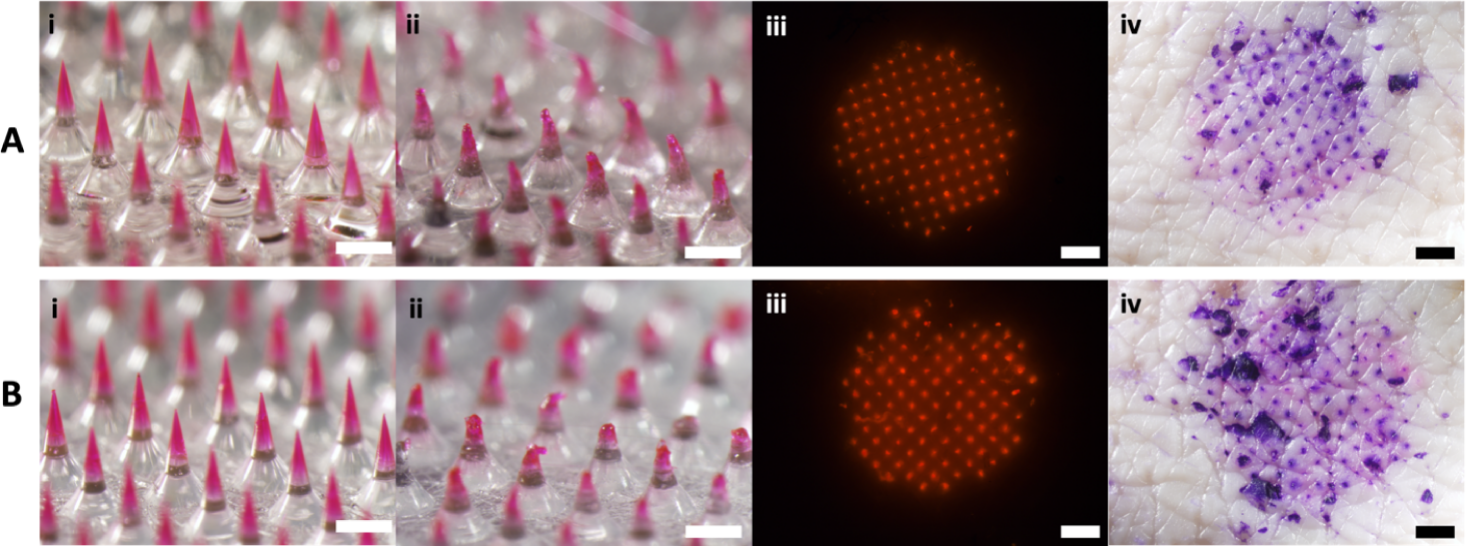
PVP MNs fabricated with aqueous and ethanol casting solutions. Representative images of MN patches made using aqueous (A) and ethanol (B) casting solutions and loaded with SRB to facilitate imaging shown before (i) and after (ii) insertion into the porcine skin ex vivo. After patch removal, skin was imaged by fluorescence microscopy (to show SRB delivered into the skin) (iii) and stained with gentian violet and imaged by brightfield microscopy (to show sites of MN puncture into the skin) (iv). Images are each representative of at least 4 replicates. Scale bars in i and ii = 500 μm; scale bars in iii and iv = 2 mm.

The objective of our work was to formulate MNs for rapid dissolution in the skin. We therefore chose to make the MNs out of PVP for a number of reasons. First, the combination of hydrophilic and hydrophobic characteristics of PVP allows MNs to be fabricated from PVP when cast in either an aqueous or organic solvent, such as ethanol. Both aqueous- and ethanol-cast PVP MNs resulted in patches with sharp and rigid MNs (Figure 1). Both patches inserted well into the porcine skin ex vivo, as shown by fluorescence imaging of the skin to visualize PVP MNs dissolved in the skin (Figure 1A(iii),B(iii)) and by gentian violet staining to show the sites where MNs penetrated into the skin (Figure 1A(iv),B(iv)). The ability to fabricate PVP MNs with either an aqueous or organic solvent demonstrates the versatility of using PVP to fabricate MNs with drugs of varying solubilities.

**Figure 2:**
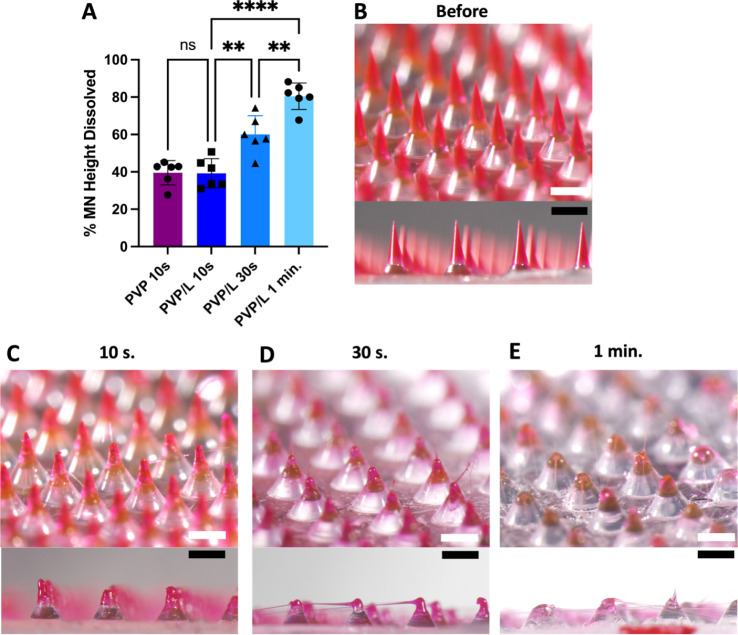
Dissolution rate of PVP MNs formulated with lidocaine. (A) Percent original height of PVP and PVP/L MNs dissolved and (B) representative images of PVP/L MNs before and 10, 30, or 60 s after insertion into the porcine skin ex vivo. In panel (A), one-way ANOVA and Tukey’s posthoc comparison determined statistical significance (**p < 0.01, **** *p* < 0.0001, *N* = 6 replicates per sample). In panel B, images are each representative of at least 6 replicates and scale bars = 500 μm.

Another advantage of fabricating MNs from PVP is its fast dissolution in water, which can enable rapid drug release and facilitate a shorter patch wear time on the skin before removal. To assess the speed of PVP MN dissolution, lidocaine was added to PVP MNs (PVP/L) by using an ethanol casting solution. We found that the addition of lidocaine to the PVP MNs did not significantly affect the rate of PVP MN dissolution (Figure 2A). Using reduction of MN height as a measure of the extent of MN dissolution, we further found that after 10 s, 30 s, or 1 min, approximately 40%, 60%, or 80% of the PVP/L MN height was dissolved, respectively, which is similar to some studies^17−19^ and faster than others using PVP MNs^20,21^ and MNs made from other water-soluble polymers.^22,23^

### 3.2 Highly Water-Soluble Salts Can Be Added to PVP MN Formulations

To further speed up the dissolution rate of PVP/L MNs, we hypothesized that adding highly water-soluble salts to the casting solution would speed up the dissolution in the skin. CA, NaB, and NaP were chosen since they are all salts found on the FDA’s Generally Regarded As Safe (GRAS) list and are commonly used excipients in pharmaceutical formulations.^24^ We also hypothesized that combining CA with NaB to yield an effervescent formulation would further speed up the MN dissolution. The generation of CO_2_ gas and subsequent convective forces from the effervescent reaction between CA and NaB could act in a similar manner to effervescent excipients used to rapidly disintegrate tablets^25^ and in previous MN patch designs.^26−28^ We also included a formulation containing CA and NaP, since NaP is a similar salt to NaB but is nonreactive with CA.

**Figure 3:**
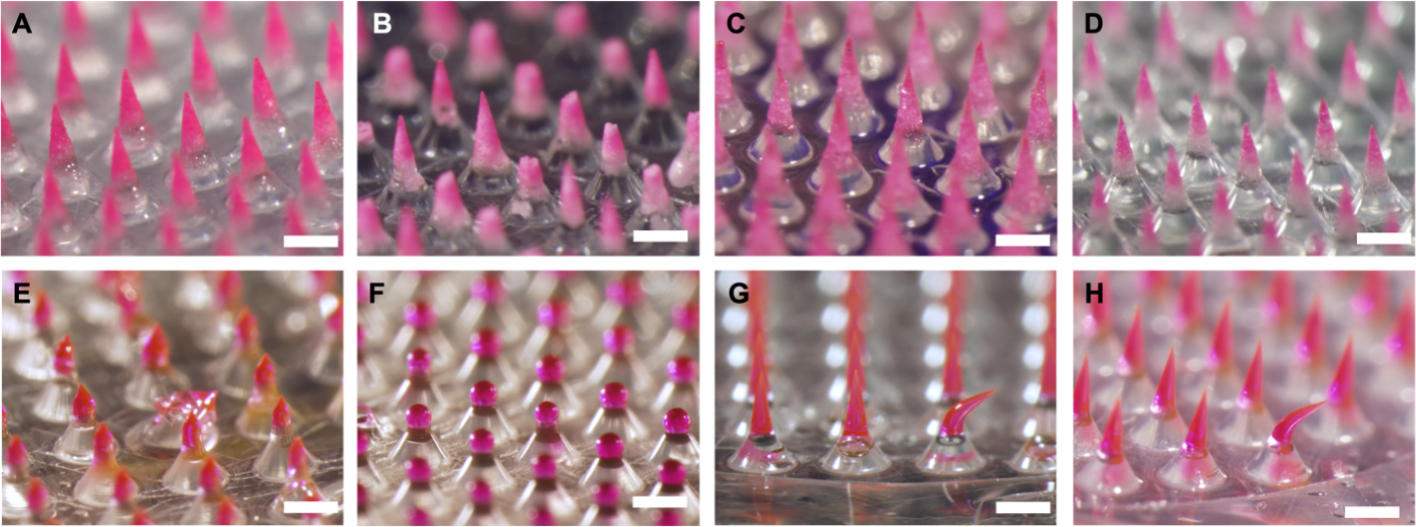
Addition of highly water-soluble salts to PVP/L MN formulation. Representative images of (A) PVP/L/NaB, (B) PVP/L/NaP, (C) PVP/L/NaB/CA, (D) PVP/L/NaP/CA, and (E) PVP/L/CA dried under 55 °C and −95 kPa vacuum after demolding. (F) PVP/L/CA dried at 55 °C without vacuum. (G) PVP/L/CA was dried at room temperature in a desiccator before demolding. (H) PVP/L/CA dried at room temperature in a desiccator after demolding. Scale bars: 500 μm. Images are representative of at least 4 replicates.

CA was soluble in the ethanol used in our casting solutions, while NaB and NaP were insoluble in ethanol and were therefore added to form a suspension in the casting solution. Using that approach, PVP MNs with lidocaine were fabricated containing these salts (Figure 3). Patches containing NaB (PVP/L/NaB) and the two effervescent formulations (PVP/L/NaB/CA and PVP/L/NaP/CA) both resulted in sharp, rigid MNs (Figure 3A,C,D). The formulation with NaP (PVP/L/NaP) also had sharp, rigid MNs (Figure 3B), but many broke off during demolding from the PDMS mold; so, this formulation was not further pursued (Figure 3D).

The CA formulation (PVP/L/CA) did not have broken MN upon demolding, but MNs were malleable and swelled after the vacuum oven drying process (Figure 3E). The MN drying process for PVP/L/CA was therefore modified to reduce the malleability of the MNs. Postdemolding drying at 55 °C without vacuum pressure resulted in MNs that completely melted and lost their shape (Figure 3F). Room temperature desiccation for 1 week before demolding (Figure 3G) or 1 week after demolding (Figure 3H) instead of vacuum oven drying still resulted in malleable MNs. The cause for the malleability of only this formulation may be due to ionic liquid formation between CA and lidocaine (Figure S2), so PVP/L/CA was not further pursued.

### 3.3 Addition of Salts Speeds Up MN Dissolution in Agarose Gel

**Figure 4:**
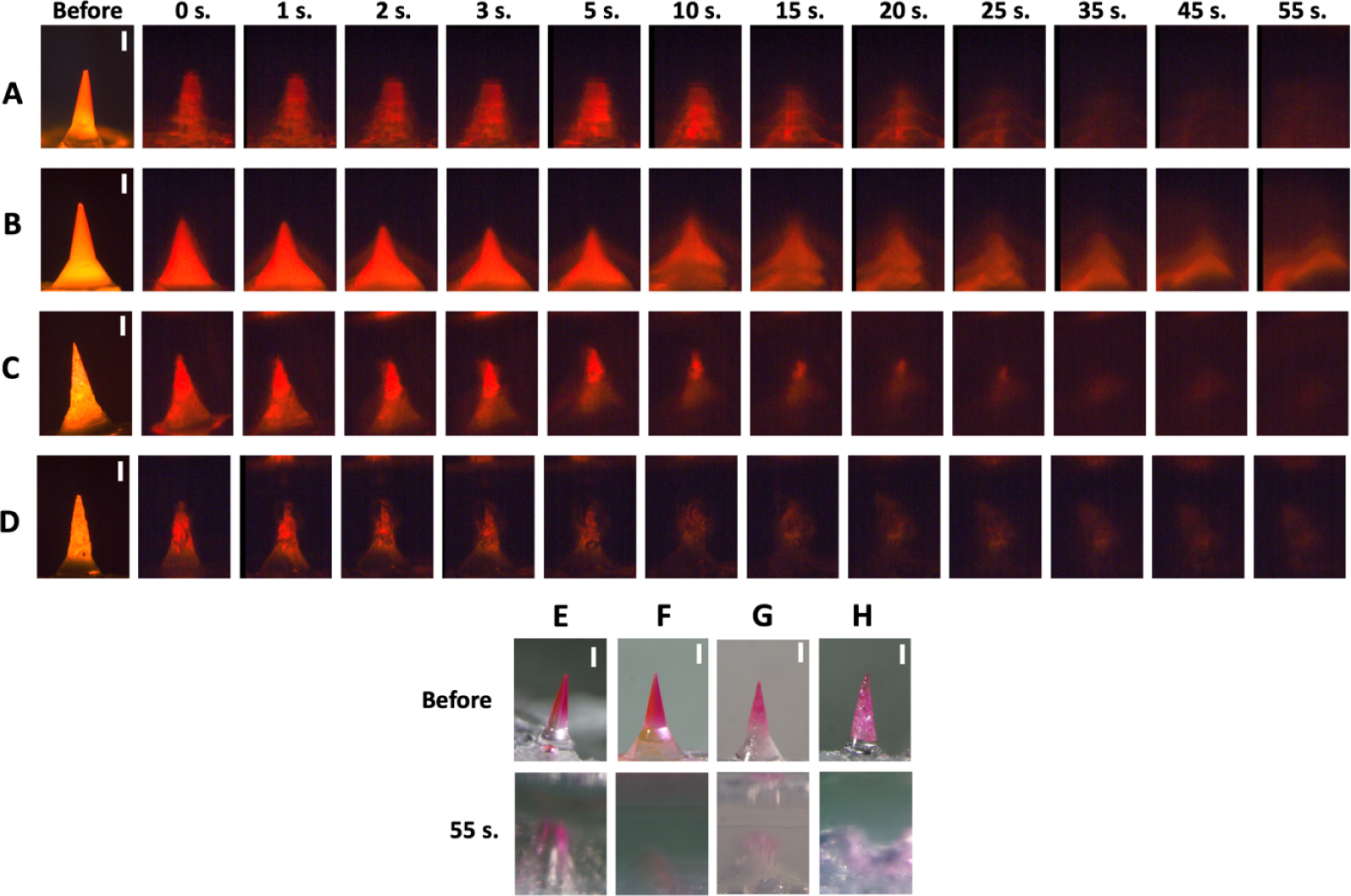
Representative images showing the effect of MN formulation on MN dissolution in agarose gel. MNs formulated with SRB in (A) PVP, (B) PVP/L, (C) PVP/L/NaB, and (D) PVP/L/NaB/CA, shown before and at various times after insertion into agarose gel and imaged with fluorescence microscopy. MNs formulated with SRB in (E) PVP, (F) PVP/L, (G) PVP/L/NaB, and (H) PVP/L/NaB/CA, shown before and at 55 s after insertion into agarose gel and imaged with brightfield microscopy. Images are each representative of at least 4 replicates. Scale bars = 200 μm.

With these salt-based formulations in hand, we next compared their dissolution rates in vitro by fluorescence microscopy (Figure 4A–D). Agarose gel was chosen as a hydrogel model for skin in order to visually compare the dissolution rates of the MNs loaded with SRB.^16^ For the PVP-only formulation, MNs dissolved (as indicated by the loss of the SRB fluorescence signal) within ∼20 s of insertion in the gel, while for PVP/L MNs, there was fluorescence for at least 55 s, likely due to the limited water solubility of the lidocaine in the MNs. When NaB was added to the formulation to increase the dissolution rate, fluorescence faded at least as quickly as the PVP-only formulation, and when CA was added to make the NaB formulation effervescent, the signal faded within a few seconds.

Agarose gel insertions were also imaged with bright-field microscopy (Figure 4E–H). The noneffervescent PVP, PVP/L, and PVP/L/NaB formulations did not have any bubble generation. The effervescent PVP/L/NaB/CA formulation had rapid bubble generation, and convective mixing was observed until at least 55 s.

### 3.4 Addition of Salts Speeds Up MN Dissolution in Porcine Skin

**Figure 5:**
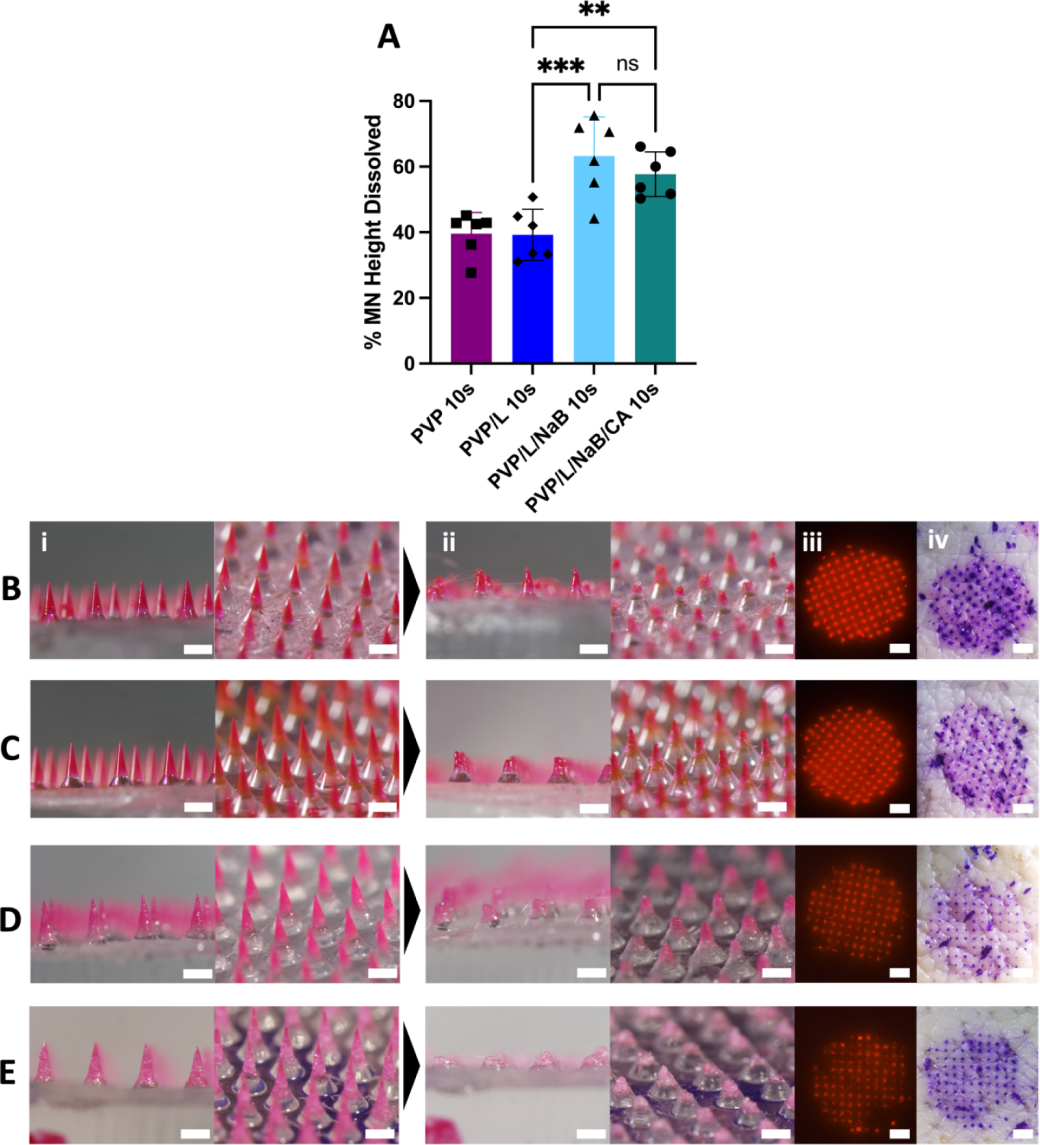
Effect of MN formulation on MN dissolution in porcine skin ex vivo. (A) Percent MN height dissolved after 10 s insertion in the skin. Representative images of (B) PVP, (C) PVP/L, (D) PVP/L/NaB, and (E) PVP/L/NaB/CA MN patches before (i) and after (ii) insertion in the skin. After insertion, skin was imaged by fluorescence microscopy (iii) and then stained with gentian violet and imaged by brightfield microscopy (iv). In panel (A), one-way ANOVA and Tukey’s posthoc comparison determined statistical significance (**p < 0.01, *** *p* < 0.001, *N* = 6 replicates per sample). In panels (B–-E), images are each representative of at least 6 replicates. Scale bars for i and ii = 500 μm and scale bars for iii and iv = 2 mm.

The PVP/L/NaB and PVP/L/NaB/CA formulations increased the dissolution rate in agarose gel, so we next investigated if they would similarly speed up dissolution when inserted into the porcine skin ex vivo. Successful MN insertion was confirmed by gentian violet staining and fluorescence imaging of the skin (Figure 5(iii),(iv)). Upon skin insertion, PVP/L/NaB had significantly more MN dissolution after 10 s insertion compared to that of PVP/L (Figure 5A), indicating the beneficial effect of the salt. Surprisingly, the effervescent PVP/L/NaB/CA MN did not have significantly more dissolution compared to the noneffervescent PVP/L/NaB (Figure 5A), indicating that effervescence did not significantly help speed up MN dissolution in skin. This may be due to the limited access to water and/or limited space for bubble growth in the skin or other factors.

### 3.5 Noneffervescent Formulation Has Better Stability Than Effervescent Formulation

**Figure 6:**
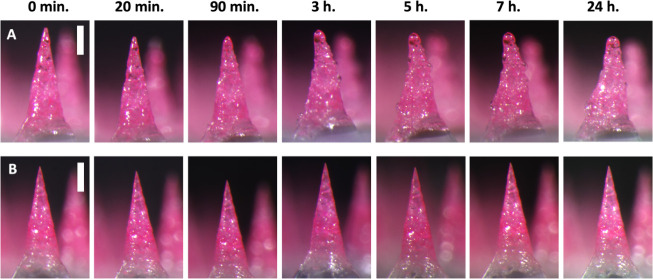
Representative images from the stability test of effervescent and noneffervescent MNs. MNs formulated with (A) effervescent PVP/L/NaB/CA or (B) noneffervescent PVP/L/Na imaged by brightfield microscopy over time during storage at room temperature and humidity. Images are each representative of at least 3 replicate patches. Scale bars = 200 μm.

To further study differences between noneffervescent PVP/L/NaB and effervescent PVP/L/NaB/CA MNs, we compared their stability during storage. We left both types of MN patches on the benchtop at ambient conditions for up to 24 h and observed changes in the MNs by microscopy. After just 90 min on the benchtop, the effervescent MNs lost their tip sharpness, and at 3 h, we see bubble formation in the MNs, presumably from prereaction of the NaB and CA (Figure 6A). Water from the atmosphere was likely absorbed by the MNs, causing them to lose their sharpness and initiate premature reaction of the effervescent excipients. CA is known to be very hygroscopic,^29^ and PVP MNs have been shown to absorb water from the atmosphere if left in high humidity.^30,31^ By contrast, the PVP/L/NaB MNs retained their shape over time and did not have bubbles formed (Figure 6B). When manipulated with forceps, the effervescent MNs had lost their rigidity and were malleable, while the PVP/L/NaB MNs did not become malleable even after 24 h. This difference in stability led us to conclude that PVP/L/NaB was the optimal formulation when considering the MN dissolution rate and long-term stability.

### 3.6 Dose Delivered by PVP/L/NaB Ex Vivo

We next used the optimized PVP/L/NaB MNs to determine the dose delivered to the skin by a patch containing 112 MNs. Measurement by HPLC showed that 23.8 ± 3.5 μg lidocaine (*n* = 4 patches) was delivered to the porcine skin ex vivo, which is well above the minimum dose expected for the analgesic effect (1.16 μg lidocaine) over the patch area (Section S1).^32,33^ The dose needed for the analgesic effect has been reported as 100 ng lidocaine/mg tissue.^33^ Our MN patch delivered ∼1052 ng of lidocaine/mg tissue, given that patches were administered to 1 cm^2^ skin, thus approximating 11.6 mg of skin as the target tissue for local analgesic effect (Section S1). Additional analysis confirmed that this lidocaine was delivered into the skin’s epidermis and dermis and not on the skin surface (Figure S3).

## 4 Discussion

This study sought to increase the speed of MN dissolution in the skin with the long-term objectives of (i) increasing the speed of drug delivery and onset of drug action for improved drug efficacy and (ii) reducing the MN patch wear time on the skin to increase patch acceptability and dosing adherence by patients. To achieve that goal, we formulated MNs with PVP due to its hydrophilic and hydrophobic properties.^13^ Its hydrophilic characteristics enable water solubility, which facilitates MN dissolution in the skin. Its hydrophobic characteristics enable dissolution in polar organic solvents like ethanol, thereby facilitating coformulation with poorly water-soluble drugs like lidocaine.

We next hypothesized that adding highly water-soluble salts to PVP MNs can further speed up the MN dissolution rate, even when coformulated with a poorly water-soluble drug like lidocaine. Adding NaB or NaB with CA to the PVP/L MN formulation allowed for ∼60% more MN dissolution in the porcine skin ex vivo compared to MNs without the salts. After just 10 s of MN insertion into skin, ∼60% of the MN height was dissolved, which allowed for the delivery of a dose of lidocaine greater than that needed for a local analgesic effect. If the MNs had been inserted for longer than 10 s, a greater percentage of the MNs would have been dissolved, and more lidocaine would have been delivered. While we sought to minimize the patch wear time on the skin to just 10 s, future studies will be needed to better understand the impact of wear time on clinical outcomes and how to balance the wear time and drug delivery dose and efficiency trade-offs. Prior studies of lidocaine delivery administered MN patches to the skin for at least 1 min,^34−36^ indicating that a 10 s wear time is a meaningful improvement.

Contrary to our initial hypothesis that effervescence would help further speed up MN dissolution, the PVP/L/NaB/CA MNs did not have any significant advantage over PVP/L/NaB when tested in the porcine skin ex vivo, although we did see faster MN dissolution in agarose gel in vitro. It could be that the rapid bubble generation upon effervescent MN insertion into the skin could have prevented access of water to the MN for dissolution due to the constrained environment in the skin that may not allow bubbles to move away from the MNs. We also found that PVP/L/NaB/CA MNs were less stable during storage conditions compared to noneffervescent PVP/L/NaB MNs. Effervescent excipients are known to be unstable in high humidity environments, as shown by those used in rapidly disintegrating tablets, which often require processing at low humidity.^37^ The ability of PVP/L/NaB MNs to maintain rigidity and sharpness in ambient storage conditions indicates that highly water-soluble excipients such as NaB but not hygroscopic excipients such as CA are good choices for MN fabrication and storage. Unlike previous studies that used effervescent MN designs to speed up release,^26−28,38^ our PVP/L/NaB MNs allow for both fast dissolution and shelf stability using noneffervescent excipients.

Interestingly, the PVP/L/CA formulation was the only one that could not form MNs rigid enough for insertion into agarose gel or skin, despite the same MN geometry and manufacturing and drying steps. We hypothesize that formation of an ionic liquid pair between the positively charged amine or amide group in lidocaine and the negatively charged acid groups of CA could explain this finding, as supported by the appearance of films made to represent the formulations (Figure S2). When cast on PDMS substrates, films made of lidocaine and CA remained as a viscous liquid droplet, consistent with ionic liquid formation, and films of PVP/L/CA were solid but very malleable. By contrast, all formulations without CA formed rigid, brittle films. The PVP/L/NaB/CA formulation formed a slightly malleable film, likely due to the addition of NaB providing enough mechanical strength to allow for a more rigid structure and successful MN fabrication.

Ionic liquid formation between lidocaine and CA was further supported by FTIR spectra (Figures S4 and S5). Characteristic bands for the pure excipients were observed at 1665 cm^–1^ (C=O in the lidocaine amide group)^39^ and 1704–1750 cm^–1^ (C=O carboxylic acid groups in CA).^39−41^ For the L/CA film, a band at 1706 cm^–1^ was instead observed, which could either indicate the presence of C=O in both lidocaine and CA in the mixture, or this relative blue shift from the 1665 cm^–1^ lidocaine peak could imply hydrogen bonding in an ionic interaction with the lidocaine amide carbonyl and CA carboxylic acid groups.^40^ The other characteristic lidocaine peak at 1478 cm^–1^ (C=C in the lidocaine aromatic ring)^42^ was no longer present in the L/CA film, indicating possible interaction or ionic liquid formation with the lidocaine tertiary amine.^43^ Excess MN water content was also ruled out as a cause for malleability in L/CA formulations, since TGA measurements showed less water content in malleable PVP/L/CA compared to nonmalleable PVP/L/NaB/CA (Figure S1).

Previous work reports higher doses of lidocaine MN delivery, which allowed for sustained analgesia and analgesia at lateral sites beyond the MN patch.^33,44−46^ Although our MN patch had a lower lidocaine dose delivered than some other studies, our patch was designed for quick, short-lasting local anesthesia. Our patch could reduce patient discomfort before performing acute treatments like sutures, catheterization, dermal biopsy, or injections and could be a more appropriate preprocedure tool, given its rapid lidocaine delivery, rather than for chronic pain management. Our MN patch is not intended for systemic or long-term pain management. Instead, it provides a less painful alternative to injected lidocaine and should offer a faster onset of action than commercial creams (∼120 min) or transdermal patches (∼60 min.) for managing discomfort during brief procedures.^44^

A limitation of our study was that we did not determine whether our MN patch contained an effective dose of lidocaine to cause local anesthesia in vivo. Our delivery of ∼24 μg lidocaine was calculated to be ∼1052 ng/mg tissue, which is above the therapeutic threshold reported in the literature (100 ng/mg tissue^33^) (Section S1). However, other literature suggests a higher range of lidocaine doses needed for the anesthetic effect, including 53 μg/cm^2^ skin,^44^ 80 μg/MN patch,^34^ 224 μg/cm^2^ skin,^45^ and up to 1 mg/patch.^46^ While our patch was loaded with 112 μg of lidocaine, which falls within these ranges, inefficient dose delivery resulted in a lower amount delivered, placing it at the lower end of the reported therapeutic range. Some of these studies reporting higher doses were also likely intended for sustained local anesthesia or over larger areas. Future in vivo studies will be needed to confirm if our dose would be effective at inducing local anesthesia for short-term pain management before performing acute procedures rather than sustained local anesthesia over a large area.

An improvement to the rapidly dissolving MN design could be the dose delivery efficiency, which was only 21.3% after a 10 s insertion in the skin, based on the mass balance calculation of lidocaine in the used patch, in the skin, and on the skin surface (Figure S3). Future work could improve the MN geometry to allow for more efficient insertion into the skin and access to fluid in the skin for raid dissolution, or the fabrication process could be optimized to more-selectively load the lidocaine toward the MN tip.

Future studies could also include formulating the PVP/NaB MN with other drugs, including poorly water-soluble drugs (e.g., benzodiazepines^47^) cast in an ethanol or other polar organic solvent or water-soluble drugs using an aqueous casting solution. Delivery of lidocaine and other drugs should be further evaluated for pharmacokinetics in animals and eventually humans and compared to other routes of administration, such as oral, topical, rectal, and injection routes. Local delivery of lidocaine to the skin with the PVP/L/NaB formulation could also be evaluated for pharmacodynamics using a method such as the paw withdrawal or tail flick test^48−50^ to determine if the local anesthetic effect of the MN patch is different from topically applied creams or injection.

## 5 Conclusion

In this study, we demonstrate the rapid dissolution of PVP MNs that was further enhanced by the addition of water-soluble salts to enable fast delivery of lidocaine, which can serve as a model drug with poor water-solubility. We show that either addition of NaB or NaB with CA (effervescent formulation) enabled significantly faster MN dissolution in the pig skin ex vivo. However, the effervescent formulation did not offer significantly faster dissolution over the NaB-only formulation and was not as shelf-stable as the NaB-only formulation, indicating that the noneffervescent formulation was preferred. The optimized PVP/L/NaB MN formulation delivered therapeutically relevant levels of lidocaine into skin and could be used for the delivery of other drugs as well that would benefit from rapid delivery into the skin with a short patch wear time.

## Supporting Information Available

A calculation of the lidocaine dose needed for analgesic effect, as well as data on the water content measured by TGA, film casts of the MN formulations, HPLC analysis of MN patch delivery efficiency, and FTIR spectra of MN formulations (PDF)
