# Supplementary material for: Highly Water-Soluble Microneedle Patch for Short Wear Time and Rapid Drug Delivery
Source: Mol Pharm. 2024 Dec 3;22(1):573–82. doi: 10.1021/acs.molpharmaceut.4c01207 (PMC11707735; doi:10.1021/acs.molpharmaceut.4c01207)
Supplement: Supplementary file 1 — mp4c01207_si_001.pdf [file mp4c01207_si_001.pdf]

Supplementary Material

Highly water-soluble microneedle patch for short wear time and rapid drug delivery

Amy J. Wood-Yang, Abishek Sankaranarayanan, Max J. Freidlin, Mark R. Prausnitz*

School of Chemical and Biomolecular Engineering, Georgia Institute of Technology, Atlanta, GA 30332, USA.

* To whom correspondence should be addressed: prausnitz@gatech.edu

Supplementary Section S1: Calculation of dose needed for analgesic effect.

Li et al.[1] found that the dermis density is 116 kg/m^3 (i.e., 0.116 g/cm^3). Zhang et al.[2] found that the minimum threshold for lidocaine content in skin required for local analgesic effect was 100 ng lidocaine/mg skin. For 0.1 cm^3 skin (based on the approximate size of the microneedle patch used in the current study: 1 cm x 1 cm patch, 0.1 cm thick),

$$0.1\ cm^3 skin \cdot \frac{0.116\ g\ skin}{1\ cm^3 skin} \cdot \frac{1000\ mg\ skin}{1\ g\ skin} = 11.6\ mg\ skin$$

$$\frac{100\ ng\ lidocaine}{1\ mg\ skin} \cdot 11.6\ mg\ skin = \boldsymbol{1160\ ng\ lidocaine\ needed\ per\ patch}$$

Supplementary References

[1] Li, C., Guan, G., Reif, R., Huang, Z. & Wang, R.K. 2012 Determining elastic properties of skin by measuring surface waves from an impulse mechanical stimulus using phase-sensitive optical coherence tomography. *J R Soc Interface* **9**, 831-841. (doi:10.1098/rsif.2011.0583).

[2] Zhang, Y., Brown, K., Siebenaler, K., Determan, A., Dohmeier, D. & Hansen, K. 2012 Development of lidocaine-coated microneedle product for rapid, safe, and prolonged local analgesic action. *Pharmaceutical research* **29**, 170-177.

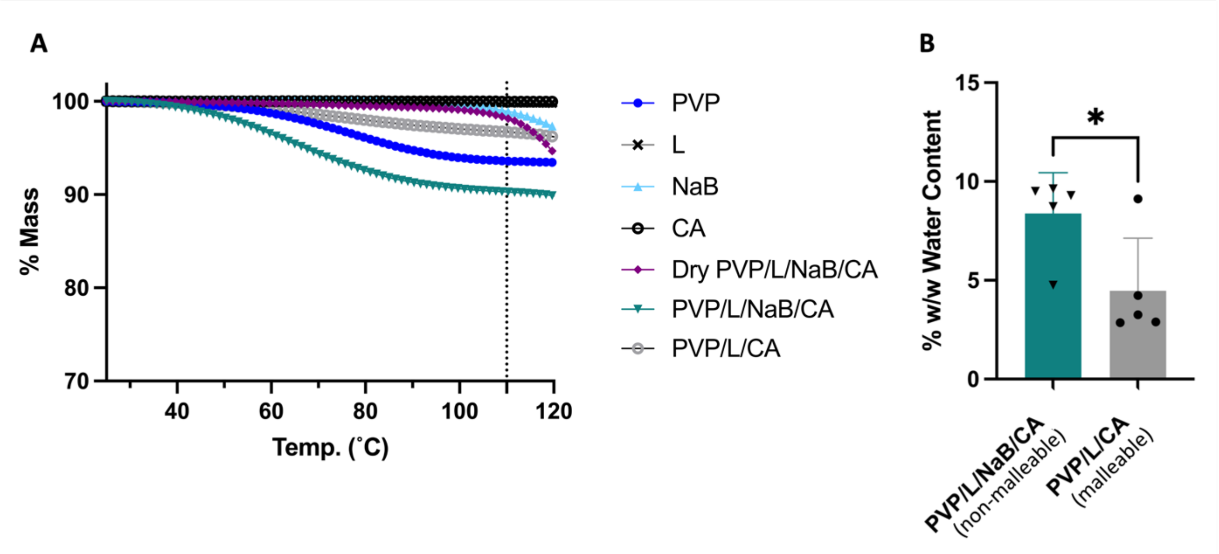

Supplementary Figure S1. TGA analysis of water content and degradation of excipient/drug film casts of MN formulations. **(A)** Representative TGA thermograms of films composed of excipient/drug mixtures and their individual components. A dry mixture of PVP/L/NaB/CA powders at the same ratios as the film-casting formulation was also included. Dotted line indicates 110˚C, since water content was calculated by taking the mass lost between 25 – 110 ˚C. **(B)** Water content in PVP/L/NaB/CA and PVP/L/CA film determined by TGA analysis (Welch's t-test, * $p < 0.05$, N = 5 replicates per sample).

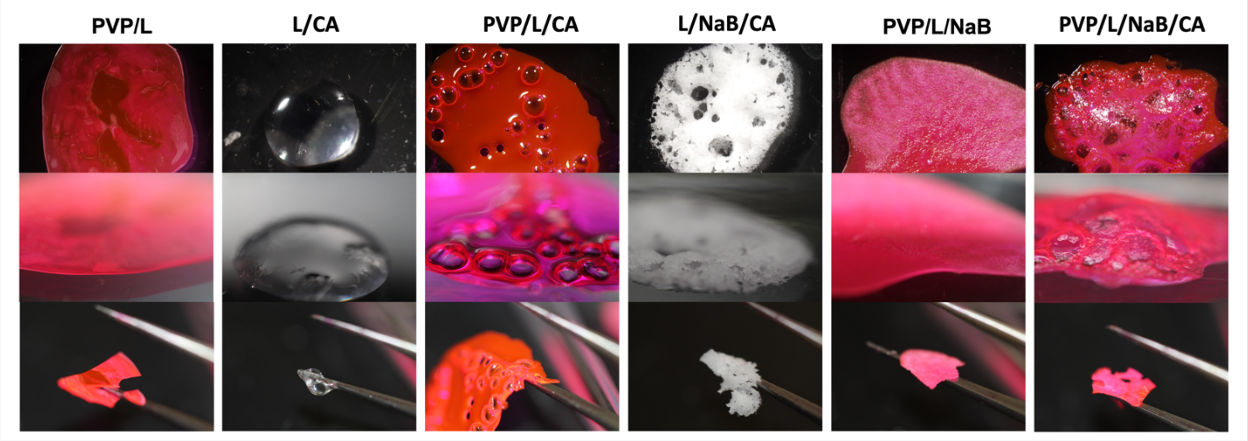

Supplementary Figure S2. Film casts of MN formulations for assessment of dried formulation mechanical properties. Representative images of formulations used for MN fabrication (**Table 1** in main manuscript) after casting onto PDMS substrates and allowed to dry. Most films appeared rigid, but PVP/L/CA was malleable, and L/CA appeared to be a liquid, suggested formation of an ionic liquid by lidocaine and citric acid.

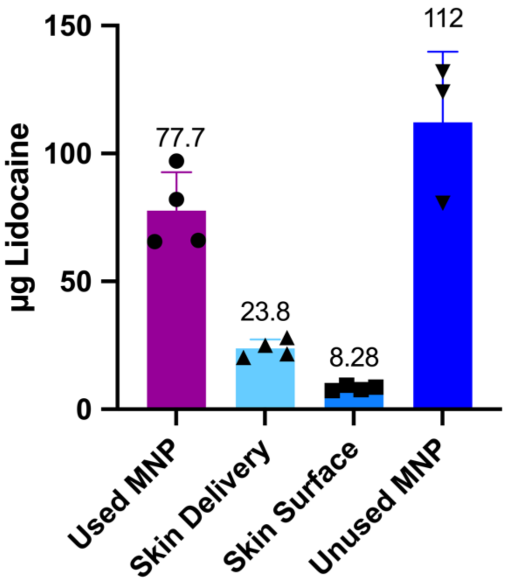

Supplementary Figure S3. HPLC analysis of MN patch delivery efficiency. The amount of lidocaine was measured in fresh MN patches, used MN patches, in the skin, and on the skin surface. The amount of lidocaine in fresh MN patches (112 µg) roughly equals the amount of lidocaine in the other three groups (110 µg), thereby closing the mass balance calculation. Skin delivery was 23.8 ± 3.5 µg, representing a 21.3% delivery efficiency relative to the fresh MN patch.

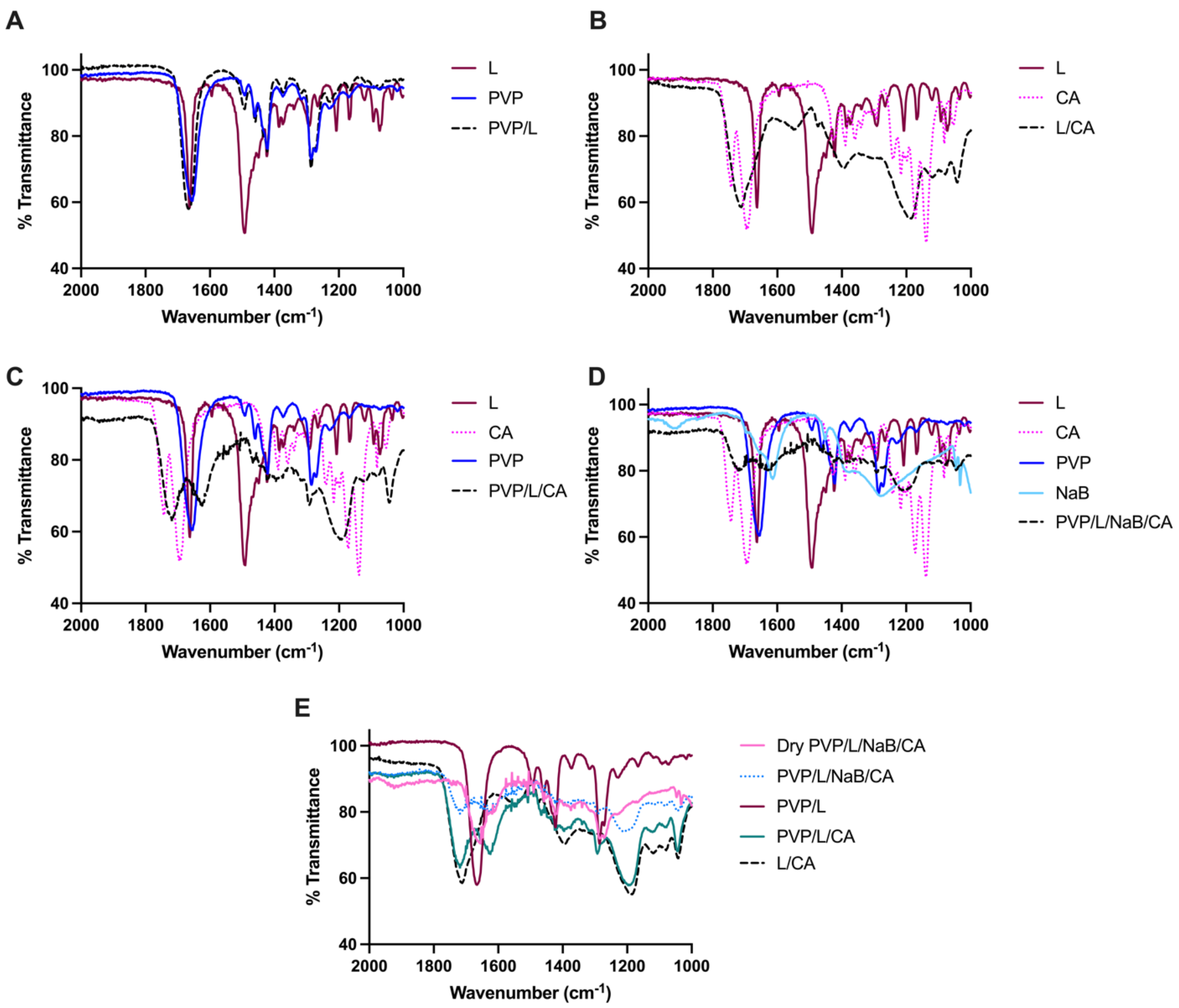

Supplementary Figure S4. FTIR spectra of film casts of MN formulations shown in Supplementary Figure 1. Representative FTIR spectra are shown for films composed of excipient/drug mixtures and their individual components on the same graph for **(A)** PVP/L, **(B)** L/CA, **(C)** PVP/L/CA, and **(D)** PVP/L/NaB/CA. **(E)** Representative FTIR spectra are also shown for the films composed different excipient/drug mixtures in parts (A) – (D). A dry mixture of PVP/L/NaB/CA powders at the same ratios as the film-casting formulation was also included.

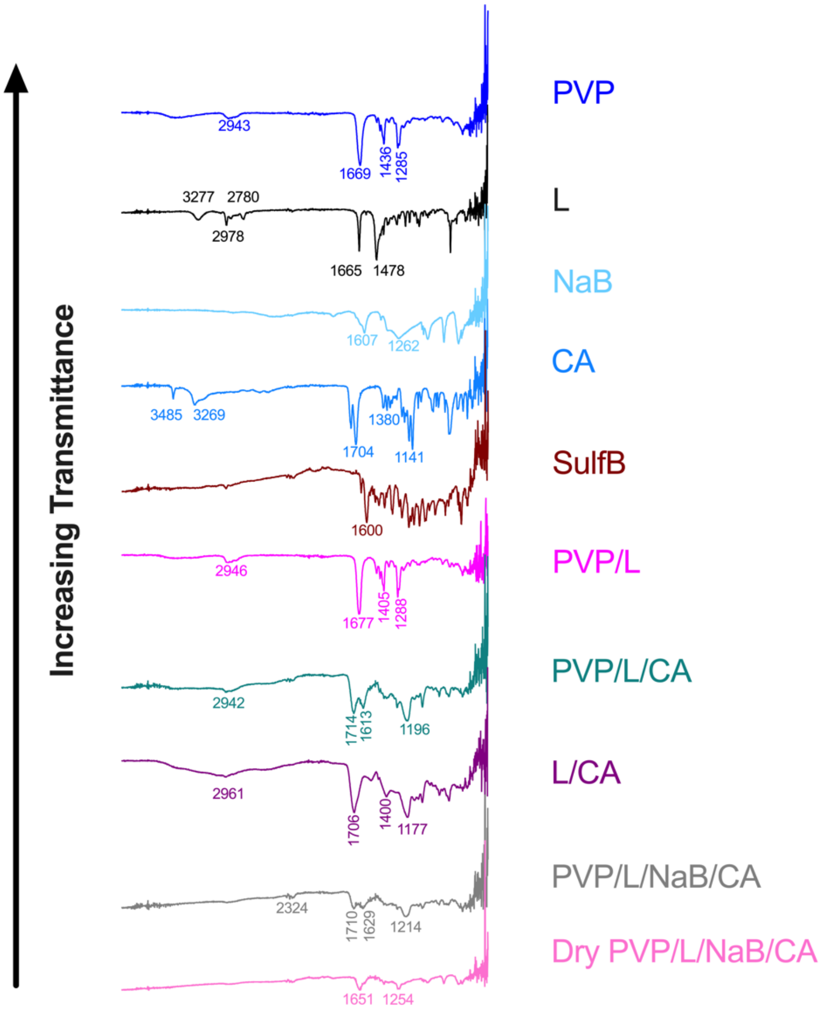

Supplementary Figure S5. FTIR spectra and band locations of film casts of MN formulations shown in Supplementary Figures S1 and S2. Representative FTIR spectra are shown with characteristic band locations identified.
